# Machine Learning for the Prediction of Acute Kidney Injury in Critically Ill Patients With Coronary Heart Disease: Algorithm Development and Validation

**DOI:** 10.2196/72349

**Published:** 2025-05-28

**Authors:** Yike Li, Mingyang Xiao, Yaqian Li, Lulu Lv, Shanshan Zhang, Yuhui Liu, Juan Zhang

**Affiliations:** 1 The Second Clinical Medical School Zhengzhou University Zhengzhou China

**Keywords:** coronary heart disease, coronary artery disease, acute kidney injury, machine learning, MIMIC-IV database

## Abstract

**Background:**

Acute kidney injury (AKI) frequently occurs in critically ill patients with coronary heart disease (CHD), and its development markedly elevates mortality rates and prolongs hospitalization duration. Early AKI prediction is crucial for timely intervention and amelioration of patient outcomes.

**Objective:**

This study aimed to develop and verify a clinical prediction model for the occurrence of AKI upon admission in the critically ill population with CHD through machine learning (ML).

**Methods:**

Data from the MIMIC-IV (Medical Information Mart for Intensive Care IV) version 2.2 database were gathered and included information about critically ill individuals with CHD in the intensive care unit (ICU). The dataset was randomized into a training set (70%) and a testing set (30%). Least absolute shrinkage and selection operator (LASSO) regression was used for feature variable selection. ML models, including logistic regression (LR), decision tree (DT), naive Bayes (NB), random forest (RF), extreme gradient boosting (XGBoost), and support vector machine (SVM), were constructed using 13 variables in the training set. The 6 models were compared in the testing set to identify the best-performing model. Subsequently, the model was assessed using calibration curve analysis and decision curve analysis (DCA). External validation was conducted using data from the Second Affiliated Hospital of Zhengzhou University. Ultimately, the predictive model was interpreted via Shapley Additive Explanation (SHAP) values.

**Results:**

In total, 2711 patients with CHD admitted to the ICU were selected, with 1809 (66.7%) having AKI. XGBoost exhibited the best performance regarding discrimination (area under the receiver operating characteristic curve [AUROC]=0.765, 95% CI 0.731-0.800), accuracy (0.725), and sensitivity (0.759). External validation using a cohort of 226 patients confirmed the strong generalizability of the XGBoost model (AUROC=0.835, 95% CI 0.782-0.887). Feature importance analyses derived from SHAP values, DT, RF, and XGBoost consistently identified 5 key predictors associated with the development of AKI: mechanical ventilation, use of antiplatelet agents, age, N-terminal pro–B-type natriuretic peptide (NT-proBNP) levels, and acute physiology score III (APSIII).

**Conclusions:**

ML models can serve as reliable tools for forecasting AKI in the critically ill population with CHD. The XGBoost model is highly accurate and may aid doctors in identifying high-risk individuals for early intervention to lower mortality.

## Introduction

Acute kidney injury (AKI) is a frequent complication in people admitted to the intensive care unit (ICU), particularly in the coronary care unit (CCU). The incidence of AKI in the CCU is 28.5% [1]. In patients in the ICU, the onset of AKI is linked to significantly longer hospital stays and higher in-hospital death rates in contrast to the population without AKI, resulting in a worse overall prognosis [2]. However, with timely intervention and effective treatment, AKI can be reversed early, thereby reducing mortality related to AKI [3]. Therefore, early AKI identification is essential for critically ill individuals with coronary heart disease (CHD) in the ICU. To better manage critically ill patients with CHD, an accurate predictive model is required to find high-risk individuals, enabling early intervention to ameliorate their prognosis.

In recent years, several cardiac disease–related AKI prediction models have been created. For instance, Ma et al [4] used nomograms to develop a prediction model for contrast-induced AKI in individuals with non–ST elevation acute coronary syndrome (ACS), and Peng et al [5] developed a machine learning (ML)-based predictive model for AKI in patients with congestive heart failure. Existing predictive models are primarily designed for patients with isolated risk factors, such as acute myocardial infarction (AMI) or those undergoing percutaneous coronary intervention (PCI). However, critically ill patients admitted to the ICU often present with complex conditions and multiple comorbidities, rendering current models less applicable to this population. Therefore, it is important to establish a more widely applicable predictive model for AKI in the critically ill cohort with CHD. As statistical theory and computational technologies advance, artificial intelligence (AI) is beginning to play a vital role in medicine. AI can learn to recognize large datasets from databases, enabling precise disease prediction, which helps clinicians develop appropriate treatment plans [6,7]. ML approaches have been extensively used in the construction of predictive models for multiple illnesses, exhibiting higher performance in comparison to conventional models, such as logistic regression (LR) or Cox regression analysis [8,9].

This study endeavored to assess the incidence of AKI following hospital admission in critically ill patients with CHD, as well as to identify potential influencing factors. Using a range of ML algorithms, we aimed to develop a generalizable predictive model for postadmission AKI in this patient population, identify the model with the best predictive performance, evaluate its accuracy and generalizability, and conduct interpretability analyses. Our model is designed to operate within an electronic health record (EHR) system to continuously monitor and analyze patient data in real time, automatically calculate the risk score for AKI, and issue early warnings in high-risk cases. This approach may assist clinicians in implementing timely preventive measures or initiating early interventions.

## Methods

### Study Design and Population

The Medical Information Mart for Intensive Care IV (MIMIC-IV) database, a single-center ICU dataset of the Laboratory for Computational Physiology at the Massachusetts Institute of Technology (MIT), has gained approval from the Institutional Review Boards (IRBs) of MIT and the Beth Israel Deaconess Medical Center (BIDMC). The patient information within the database is anonymized, obviating the need for informed consent from patients [10,11]. MIMIC-IV version 2.2 includes medical data from 73,181 patients in the ICU from 2008 to 2019 [12]. Our study cohort consisted of patients diagnosed with CHD upon admission. The inclusion criteria were (1) age of 18 years or more; (2) hospitalization duration exceeding 24 hours; (3) patients diagnosed with CHD according to the *International Classification of Diseases, 9th Revision* (ICD-9: 41401, 41402, 41404, 41405, 41407) and *International Classification of Diseases, 10th Revision* (ICD-10: I2101, I2102, I2109, I2111, I2119, I2121, I240, I251). In total, 30,136 patients with CHD were found in the MIMIC-IV database. Patients were excluded if they (1) were not admitted to the ICU (n=13,461); (2) had missing outcome variables (n=1); (3) had missing categorical variables, such as gender, marital status, and ethnicity (n=2420); (4) had missing N-terminal pro–B-type natriuretic peptide (NT-proBNP) or neutrophil count data (n=11,430); (5) had a history of pre-existing renal disease (n=99); or (6) had a lymphocyte count of 0 (n=14).

Additionally, clinical data were collected from 226 critically ill patients with CHD admitted to the Second Affiliated Hospital of Zhengzhou University from January 1, 2021, to August 1, 2024, as an external validation cohort. A retrospective analysis was performed using the hospital’s EHR system. The eligibility criteria were consistent with those for the training and internal validation cohorts.

### Outcome

Our research team was granted access to the MIMIC-IV database on July 12, 2024, and subsequently extracted data on 2711 critically ill patients with CHD admitted to the ICU. As of March 30, 2025, we had also collected external validation data from 226 patients at the Second Affiliated Hospital of Zhengzhou University. A total of 7 investigators participated in this study.

### Data Extraction and Processing

Data extraction was completed via structured query language (SQL) and the PostgreSQL tool (version 16.0). The extracted data consisted of the first recorded vital signs and laboratory parameters following ICU admission, as well as medications administered within the first 7 days of hospitalization. The study extracted the following variables:

- Demographic characteristics: gender, age, race, blood pressure, and BMI
- Comorbidities: AMI, history of previous myocardial infarction (MI), diabetes mellitus, hypertension, heart failure, and atrial fibrillation
- Vital signs: systolic blood pressure, diastolic blood pressure, heart rate, and respiratory rate
- Laboratory parameters: white blood cells (WBCs), red blood cells (RBCs), platelets, albumin, hemoglobin, aspartate aminotransferase (AST), alanine aminotransferase (ALT), lymphocyte percentage, lymphocyte count, neutrophil percentage, neutrophil count, neutrophil-to-lymphocyte ratio (NLR), anion gap, bicarbonate, total bilirubin, serum potassium, serum sodium, NT-proBNP, blood urea nitrogen (BUN), serum chloride, creatine kinase isoenzymes (CKMB), international normalized ratio (INR), lactate dehydrogenase (LDH), prothrombin time (PT), activated partial thromboplastin time (PTT), RBC distribution width (RDW), and serum creatinine (SCr)
- Medication use and treatment: antiplatelet agents, antihypertensive drugs, statins, vasopressors, heparin, diuretics, coronary angiography (CAG), or PCI
- Simplified acute physiology score III (APSIII) and sequential organ failure assessment (SOFA)

Variables having more than 20% missing values were eliminated from the final cohort in order to reduce bias brought on by missing data. Other variables with missing data were imputed via multiple imputation, a widely used and effective technique to handle missing data [13] estimating multiple plausible values for each missing entry [14]. Additionally, to enhance the accuracy and reliability of data and models, outliers were identified using box plots [15], and extreme values were mitigated through winsorization [16], wherein data points above the 99th percentile were substituted with the value at the 99th percentile and those below the 1st percentile were replaced with the value at the 1st percentile.

### Outcome Variables

Our study cohort comprised adult patients diagnosed with AKI following hospital admission. The diagnosis of AKI was based on the guidelines published in 2012 by the organization called Kidney Disease: Improving Global Outcomes (KDIGO) [17]. AKI was defined by the presence of any one of the following criteria: (1) an increase in SCr by ≥26.5 μmol/L (≥0.3 mg/dL) within 48 hours; (2) an increase in SCr to >1.5 times the baseline value, which is known or presumed to have occurred within the prior 7 days; or (3) a urine output of <0.5 mL/kg/hour for more than 6 hours.

### Variable Selection

To minimize the potential bias of multicollinearity and model overfitting, we used the least absolute shrinkage and selection operator (LASSO) regression to select and filter variables in the training dataset. LASSO is a regression-based method that reduces model complexity through the construction of a penalty function. The optimal regularization parameter (λ) was determined using tenfold cross-validation, and variables with nonzero coefficients were retained as the final predictors. Following LASSO regression, the variance inflation factor (VIF) was calculated for the included variables to assess multicollinearity. The VIF values for all predictors are presented in Table S1 in Multimedia Appendix 1.

### Model Development and Evaluation

The sample was randomized into a training set and a testing set in a 7:3 ratio. Six ML models were established in the training set: LR, decision tree (DT), naive Bayes (NB), random forest(RF), extreme gradient boosting (XGBoost), and support vector machine (SVM). To minimize overfitting and achieve optimal model performance, the hyperparameters of the ML models were tuned using a grid search. In contrast, the LR model was implemented using its conventional parameter settings. Detailed parameter configurations for each model are presented in Table S2 in Multimedia Appendix 1.

The models’ predictive performances were evaluated on the testing dataset by comparing the area under the receiver operating characteristic curve (AUROC), accuracy, precision, sensitivity, and specificity. Among these, AUROC was considered the primary performance metric. The model demonstrating the highest predictive performance was selected as the optimal model for this study. A calibration curve was subsequently plotted to assess the agreement between observed outcomes and predicted probabilities. In addition, decision curve analysis (DCA) was conducted to evaluate the clinical utility of the model. Finally, external validation was performed using data from the Second Affiliated Hospital of Zhengzhou University to assess the model’s generalizability and applicability in an independent cohort.

### Model Interpretation

To interpret the predictive models, feature importance was visualized for 3 tree-based models (DT, RF, and XGBoost) and interpreted using Shapley Additive Explanation (SHAP) values. Feature importance, quantified through each model’s intrinsic evaluation mechanism, directly reflects the contribution of individual input variables to the model’s predictive output, thereby elucidating the practical relevance of each feature in the decision-making process. SHAP has been widely leveraged to explain the contribution of predictive variables [18,19] to model output by offering consistent, locally accurate attribution values for every characteristic in the model. Higher values would suggest an elevated likelihood of AKI. SHAP dependence plots for the top 5 feature variables were generated to illustrate the relationship between the SHAP values and feature values, as well as to demonstrate feature interactions. In addition, to visually illustrate the decision-making logic of the model, a SHAP force plot for individual samples was generated to demonstrate the contribution of each variable to the model’s prediction.

### Statistical Analysis

All analyses were conducted in R version 4.3.1 (R Foundation for Statistical Computing). For continuous variables, the Shapiro-Wilk test was performed to evaluate the normality of the data. The mean (SD) was used for expressing variables having a normal distribution, and the independent samples *t* test was performed for comparison. The Wilcoxon rank-sum test was applied to compare non-normally distributed variables, which were displayed as medians (IQRs). The chi-square or the Fisher exact test was carried out to compare categorical variables, which were presented as numbers and percentages. *P*<.05 suggested statistical significance.

### Ethical Considerations

The MIMIC-IV database was approved by the IRBs of the MIT(0403000206) and the BIDMC(2011-P-001418). As all patient data contained within the database are deidentified, individual informed consent was not required. This study has obtained access authorization to the MIMIC-IV database through PhysioNet approval (ID: 13470927). In addition, the study protocol was approved by the Ethics Committee of the Second Affiliated Hospital of Zhengzhou University (approval numer: KY2024215). Informed consent was obtained from all patients enrolled at this institution, and all data were fully deidentified. Given that the study did not involve direct interaction with participants, no compensation was provided.

## Results

### Patient Characteristics

**Figure 1:**
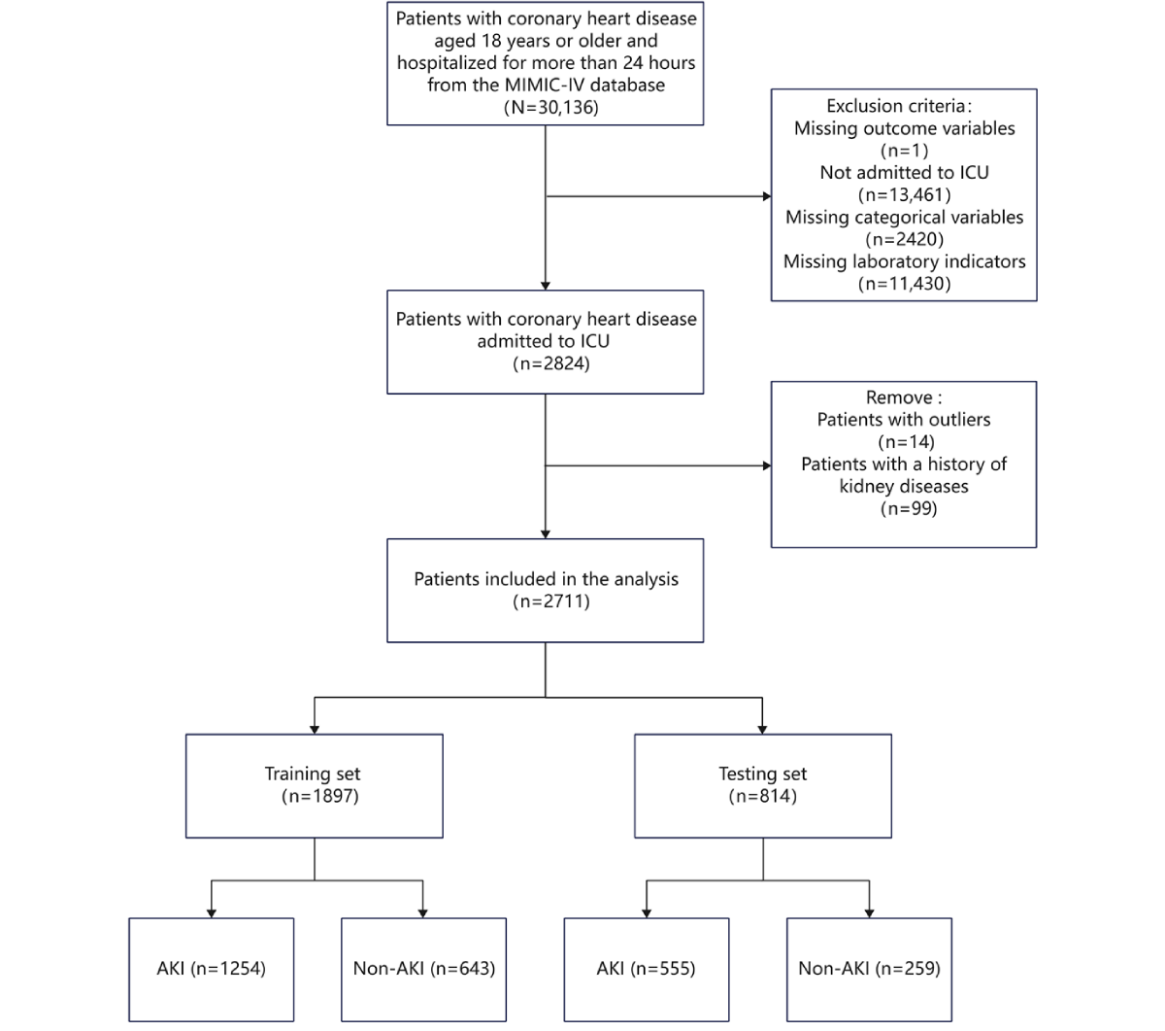
Participant-screening process diagram. AKI: acute kidney injury; ICU: intensive care unit; MIMIC-IV: Medical Information Mart for Intensive Care IV.

The inclusion process is illustrated in Figure 1. Of the 2711 patients included whose median age was 71 years, 1629 (60.1%) were males, 1985 (73.2%) White, and 1809 (66.7%) cases of AKI. After randomly splitting the dataset into a 7:3 ratio, the training group had 1897 (70%) patients, with a median age of 71 years, 1139 (60%) males, 1386 (73.1%) White, and 1254 (66.1%) cases of AKI. The testing group comprised 814 (30%) patients, with a median age of 71 years, 490 (60.2%) males, 599 (73.6%) White, and 555 (68.2%) cases of AKI. In the training set, 37 variables, including age, race, mechanical ventilation, albumin, and potassium levels, were statistically significant (*P*<.05). Table 1 presents the baseline characteristics of the patients in the training set, while the baseline characteristics of the testing set can be found in Table S3 in Multimedia Appendix 2.

**Table 1:** Baseline characteristics of the training cohort (n=1897).

Characteristics		Total patients (N=2711)	Training set (n=1897)			
			Non-AKI^a^ (n=643)	AKI (n=1254)	*P* value	
Age (years), median (IQR)		71.0 (62.0-79.0)	69.0 (60.0-79.0)	71.0 (62.0-78.0)	.02	
BMI (kg/m^2^), median (IQR)		29.1 (25.5-34.0)	29.0 (25.2-34.4)	29.0 (25.4-34.0)	.96	
**Gender, n (%)**						.08
	Male	1629 (60.1)	354 (55.1)	785 (62.6)	—^b^	
	Female	1082 (39.9)	289 (44.9)	469 (37.4)	—	
**Marital status, n (%)**						.45
	Single	661 (24.4)	170 (26.4)	307 (24.5)	—	
	Divorced/widowed	615 (22.7)	151 (23.5)	281 (22.4)	—	
	Married	1435 (52.9)	322 (50.1)	666 (53.1)	—	
**Race, n (%)**						.02
	White	1985 (73.2)	448 (69.7)	938 (74.8)	—	
	Non-White	726 (26.8)	195 (30.3)	316 (25.2)	—	
Heart rate (beats/minute), median (IQR)		83.0 (73.0-96.0)	83.0 (73.0-97.0)	82.5 (73.0-95.0)	.35	
Mechanical ventilation, n (%)		1517 (55.9)	263 (40.9)	809 (64.5)	<.001	
Respiratory rate (beats/minute), median (IQR)		18.0 (15.0-22.0)	19.0 (15.0-23.0)	18.0 (15.0-22.0)	.05	
**Laboratory values,** **median (IQR)**						
	Albumin (g/dL)	3.50 (3.00-4.00)	3.60 (3.10-4.10)	3.45 (3.00-3.90)	.001	
	ALT^c^ (IU/L)	22.0 (14.0-38.0)	23.0 (14.0-38.0)	22.0 (14.0-37.0)	.44	
	Anion gap (mmol/L)	14.0 (12.0-170)	14.0 (12.0-17.0)	14.0 (12.0-17.0)	.32	
	AST^d^ (IU/L)	29.0 (20.0-51.0)	27.0 (19.5-47.0)	29.0 (20.0-54.0)	.08	
	Bicarbonate (mmol/L)	23.0 (21.0-26.0)	23.0 (21.0-26.0)	23.0 (20.0-25.0)	.01	
	Bilirubin total (mg/dL)	0.5 (0.3-0.9)	0.5 (0.3-0.8)	0.5 (0.4-0.8)	.12	
	Potassium (mEq/L)	4.2 (3.8-4.6)	4.1 (3.8-4.6)	4.2 (3.9-4.7)	.01	
	Sodium (mEq/L)	138 (136-141)	138 (136-141)	138 (135-141)	.50	
	NT-proBNP^e^ (pg/mL)	2422 (720-6896)	1612 (470-4696)	3096 (936-8121)	<.001	
	BUN^f^ (mg/dL)	22.0 (15.0-35.0)	21.0 (14.0-32.0)	23.0 (16.0-37.0)	<.001	
	Chloride (mEq/L)	103 (100-108)	104 (99.0-107)	103 (99.0-108)	.63	
	CKMB^g^ (ng/mL)	4.0 (2.0-7.0)	3.00 (2.0-6.0)	4.0 (2.0-8.0)	<.001	
	Hemoglobin (g/dL)	10.3 (8.70-11.9)	10.4 (8.70-12.1)	10.3 (8.70-11.8)	.43	
	INR^h^	1.3 (1.1-1.5)	1.2 (1.1-1.5)	1.3 (1.1-1.5)	.13	
	Lactate (mmol/L)	1.6 (1.2-2.4)	1.7 (1.2-2.4)	1.6 (1.2-2.4)	.26	
	Lymphocyte count (10^9^/L)	1.28 (0.84-1.88)	1.32 (0.90-1.94)	1.30 (0.83-1.89)	.14	
	Lymphocytes (%)	14.1 (8.60-21.7)	15.5 (9.45-22.8)	13.6 (8.30-21.2)	.002	
	Neutrophil count (10^9^/L)	6.41 (4.34-9.74)	5.94 (4.09-8.94)	6.58 (4.51-10.1)	<.001	
	Neutrophils (%)	75.6 (67.0-83.1)	73.8 (65.8-81.7)	76.1 (67.5-83.2)	.003	
	Platelets (K/uL)	185 (138-240)	187 (136-246)	185 (137-239)	.79	
	NLR^i^	4.90 (2.90-9.10)	4.50 (2.70-7.30)	5.20 (2.90-9.57)	<.001	
	PT^j^ (seconds)	13.9 (12.3-16.5)	13.8 (12.2-16.3)	13.9 (12.4-16.5)	.16	
	PTT^k^ (seconds)	31.3 (27.6-38.5)	30.9 (27.5-37.8)	31.5 (27.7-39.7)	.09	
	RBC^l^ (m/uL)	3.50 (2.97-4.03)	3.53 (3.01-4.08)	3.46 (2.96-4.01)	.18	
	RDW^m^ (%)	14.6 (13.6-15.9)	14.6 (13.6-15.9)	14.4 (13.5-15.9)	.28	
	SCr^n^ (mg/dL)	1.1 (0.8-1.6)	1.0 (0.8-1.5)	1.1 (0.9-1.7)	<.001	
	WBC^o^ (K/uL)	10.1 (7.40-14.0)	9.50 (7.00-13.4)	10.5 (7.70-14.2)	<.001	
**Comorbidities, n (%)**						
	AMI^p^	1105 (40.8)	169 (26.3)	590 (47)	<.001	
	Atrial fibrillation	1538 (56.7)	315 (49)	747 (59.6)	<.001	
	Diabetes	1561 (57.6)	346 (53.8)	759 (60.5)	.006	
	Heart failure	2252 (83.1)	497 (77.3)	1078 (86)	<.001	
	Hypertension	2558 (94.4)	609 (94.7)	1185 (94.5%)	.93	
	Old MI^q^	1254 (46.3)	213 (33.1)	656 (52.3)	<.001	
**Drugs, n (%)**						
	Angiotensin-converting enzyme (ACE) inhibitor–angiotensin receptor blocker (ACEI-ARB)	1262 (46.6)	281 (43.7)	611 (48.7)	.04	
	Antibiotic	1928 (71.1)	388 (60.3)	935 (74.6)	<.001	
	Antiplatelet drug	2148 (79.2)	407 (63.3)	1087 (86.7)	<.001	
	Aspirin	1747 (64.4)	319 (49.6)	894 (71.3)	<.001	
	Clopidogrel	602 (22.2)	78 (12.1)	350 (27.9)	<.001	
	Dual-antiplatelet therapy	504 (18.6)	64 (10)	293 (23.4)	<.001	
	Dobutamine	121 (4.5)	17 (2.6)	74 (5.9)	.002	
	Dopamine	144 (5.31)	30 (4.7)	78 (6.2)	.20	
	Epinephrine	332 (12.2)	44 (6.8)	189 (15.1)	<.001	
	Heparin	947 (34.9)	149 (23.2)	519 (41.4)	<.001	
	Hydragogue	2172 (80.1)	451 (70.1)	1065 (84.9)	<.001	
	Noradrenaline	865 (31.9)	132 (20.5)	476 (38)	<.001	
	Two vasoactive drugs	258 (9.5)	32 (5)	140 (11.2)	<.001	
	Three vasoactive drugs	63 (2.3)	7 (1.1)	42 (3.3)	.005	
	Tatin	2138 (78.9)	429 (66.7)	1064 (84.8)	<.001	
CAG^r^, n (%)		152 (5.6)	30 (4.7)	78 (6.2)	.20	
PCI^s^, n (%)		76 (2.8)	7 (1.1)	48 (3.8)	.001	
APSIII^t^, mean (SD)		43.5 (17.0)	40.3 (16.1)	45.2 (17.4)	<.001	
SOFA^u^, median (IQR)		4.00 (2.00-6.00)	4.00 (2.00-6.00)	4.00 (2.00-7.00)	<.001	

^a^AKI: acute kidney injury.

^b^Not applicable.

^c^ALT: alanine aminotransferase.

^d^AST: aspartate aminotransferase.

^e^NT-proBNP: N-terminal pro–B-type natriuretic peptide.

^f^BUN: blood urea nitrogen.

^g^CKMB: creatine kinase isoenzymes.

^h^INR: international normalized ratio.

^i^NLR: neutrophil-to-lymphocyte ratio.

^j^PT: prothrombin time.

^k^PTT: partial thromboplastin time.

^l^RBC: red blood cell.

^m^RDW: red blood cell distribution width.

^n^SCr: serum creatinine.

^o^WBC: white blood cell.

^p^AMI: acute myocardial infarction.

^q^MI: myocardial infarction.

^r^CAG: coronary angiography.

^s^PCI: percutaneous coronary intervention.

^t^APSIII: acute physiology score III.

^u^SOFA: sequential organ failure assessment.

### Variable Selection

Based on the feature selection results from the LASSO regression (Figure S1 in Multimedia Appendix 3), 13 features were included: age, mechanical ventilation, NT-proBNP, NLR, AMI, history of prior MI, antiplatelet therapy, dual-antiplatelet therapy, heparin, diuretics, norepinephrine, statins, and APSIII.

### ML Model Performance

**Figure 2:**
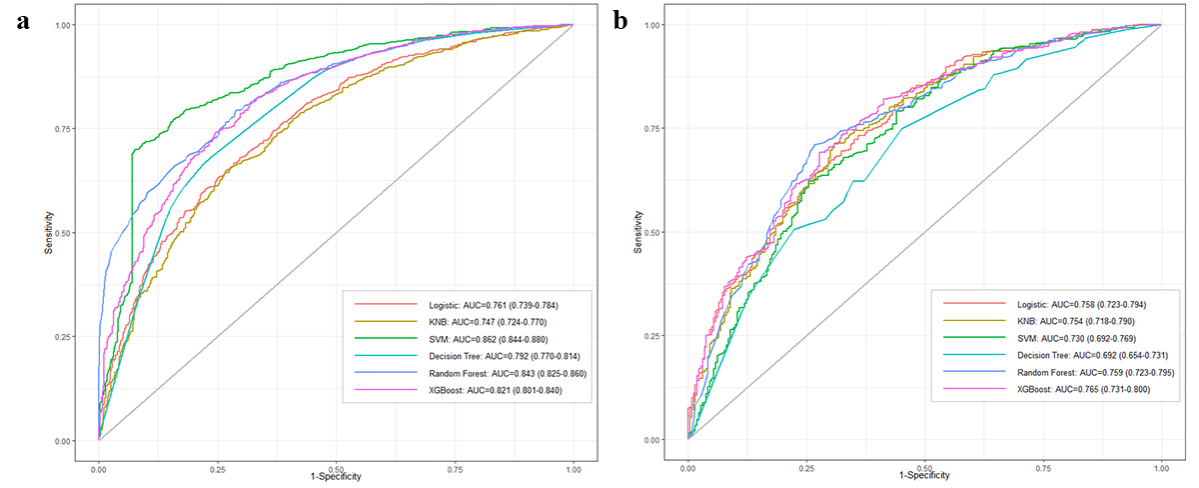
Comparison of AUC values across 6 models: (a) training set and (b) testing set. AUC: area under the curve; KNB: kernel naive Bayes; SVM: support vector machine; XGBoost: extreme gradient boosting.

Six ML models were created to forecast AKI occurrence. Figure 2 illustrates the ROC performance of these models in both training and testing cohorts. In the testing cohort, the XGBoost model (AUROC=0.765) demonstrated the best forecasting performance, followed by LR (AUROC=0.758), NB (AUROC=0.754), RF (AUROC=0.759), SVM (AUROC=0.731), and DT (AUROC=0.692). Table 2 presents detailed performance metrics for all 6 models. The XGBoost model demonstrated superior discriminative ability, with accuracy (0.725) and sensitivity (0.759) higher than those of the other 5 models, indicating that XGBoost is the optimal model.

**Table 2:** Performance indicators of 6 models.

Model and datasets		AUROC^a^	Accuracy	Precision	Sensitivity	Specificity
**LR^b^**						
	Training set	0.761	0.687	0.817	0.679	0.703
	Testing set	0.758	0.720	0.795	0.537	0.840
	External validation set	0.823	0.801	0.775	0.782	0.816
**NB^c^**						
	Training set	0.747	0.679	0.822	0.658	0.722
	Testing set	0.754	0.706	0.832	0.714	0.691
	External validation set	0.780	0.761	0.753	0.693	0.816
**SVM^d^**						
	Training set	0.862	0.775	0.947	0.699	0.924
	Testing set	0.731	0.661	0.839	0.622	0.745
	External validation set	0.786	0.761	0.733	0.733	0.784
**DT^e^**						
	Training set	0.792	0.709	0.849	0.681	0.764
	Testing set	0.692	0.686	0.780	0.750	0.548
	External validation set	0.771	0.770	0.747	0.733	0.800
**RF^f^**						
	Training set	0.843	0.766	0.843	0.794	0.711
	Testing set	0.759	0.717	0.851	0.710	0.734
	External validation set	0.771	0.752	0.747	0.673	0.816
**XGBoost^g^**						
	Training set	0.821	0.769	0.845	0.796	0.715
	Testing set	0.765	0.725	0.824	0.759	0.653
	External validation set	0.835	0.788	0.757	0.772	0.800

^a^AUROC: area under the receiver operating characteristic curve.

^b^LR: logistic regression.

^c^NB: naive Bayes.

^d^SVM: support vector machine.

^e^DT: decision tree.

^f^RF: random forest.

^g^XGBoost: extreme gradient boosting.

**Figure 3:**
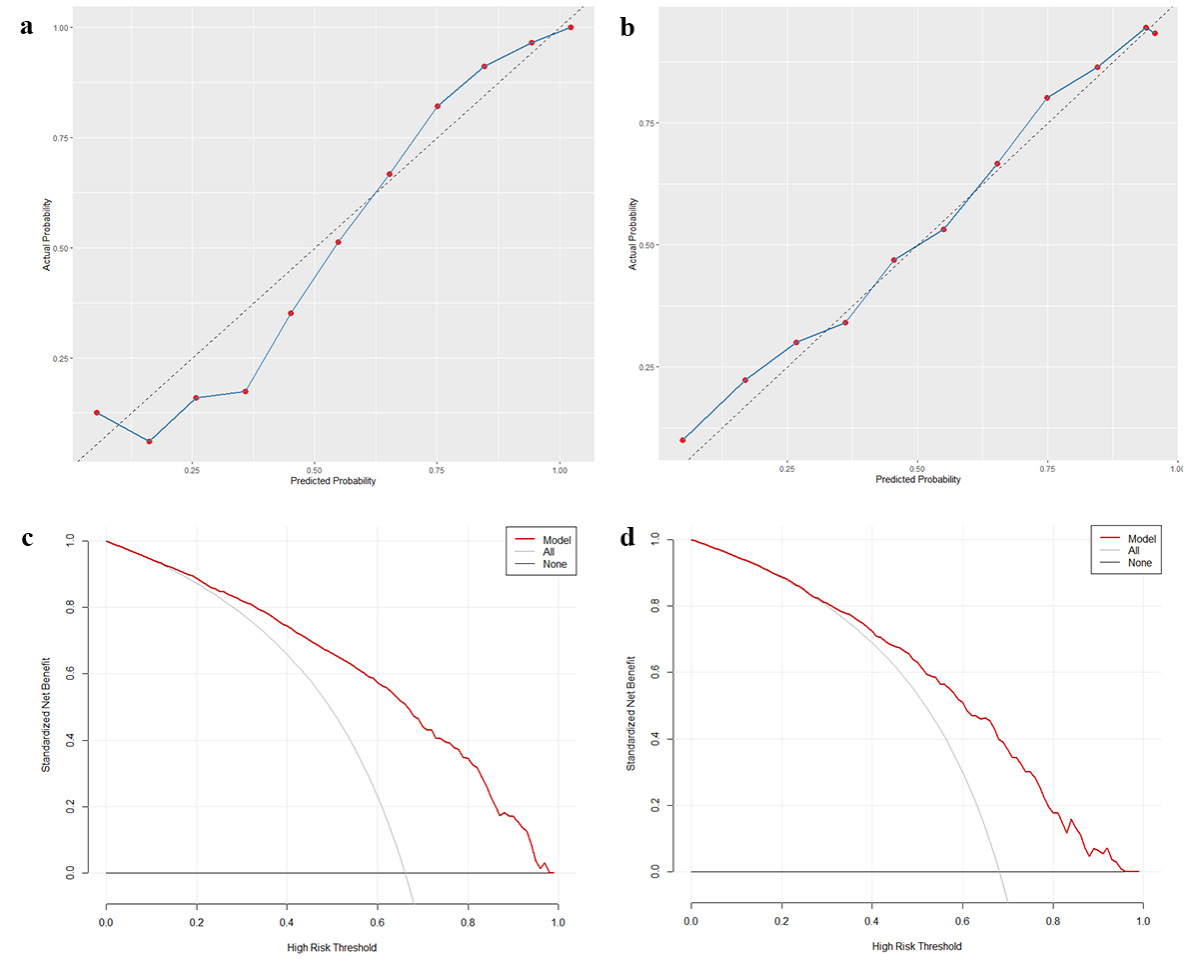
Calibration curve of the XGBoost model: (a) training set and (b) testing set. DCA of the XGBoost model: (c) training set and (d) testing set. DCA: decision curve analysis; XGBoost: extreme gradient boosting.

Calibration curves and DCA were used to further assess the ideal model (Figure 3). The calibration curve is an important tool for assessing the performance of predictive models, as it compares the predicted probabilities with actual observed outcomes to measure accuracy and reliability. Clinical DCA evaluates the practical value of a clinical prediction model by comparing the net benefit of different decision thresholds. Based on the results, the calibration curve of the testing set demonstrated excellent calibration of the model, while DCA indicated that the model provides clinical net benefit across a threshold probability range of 0.20-0.95, suggesting that the model has outstanding clinical applicability.

### External Validation

**Figure 4:**
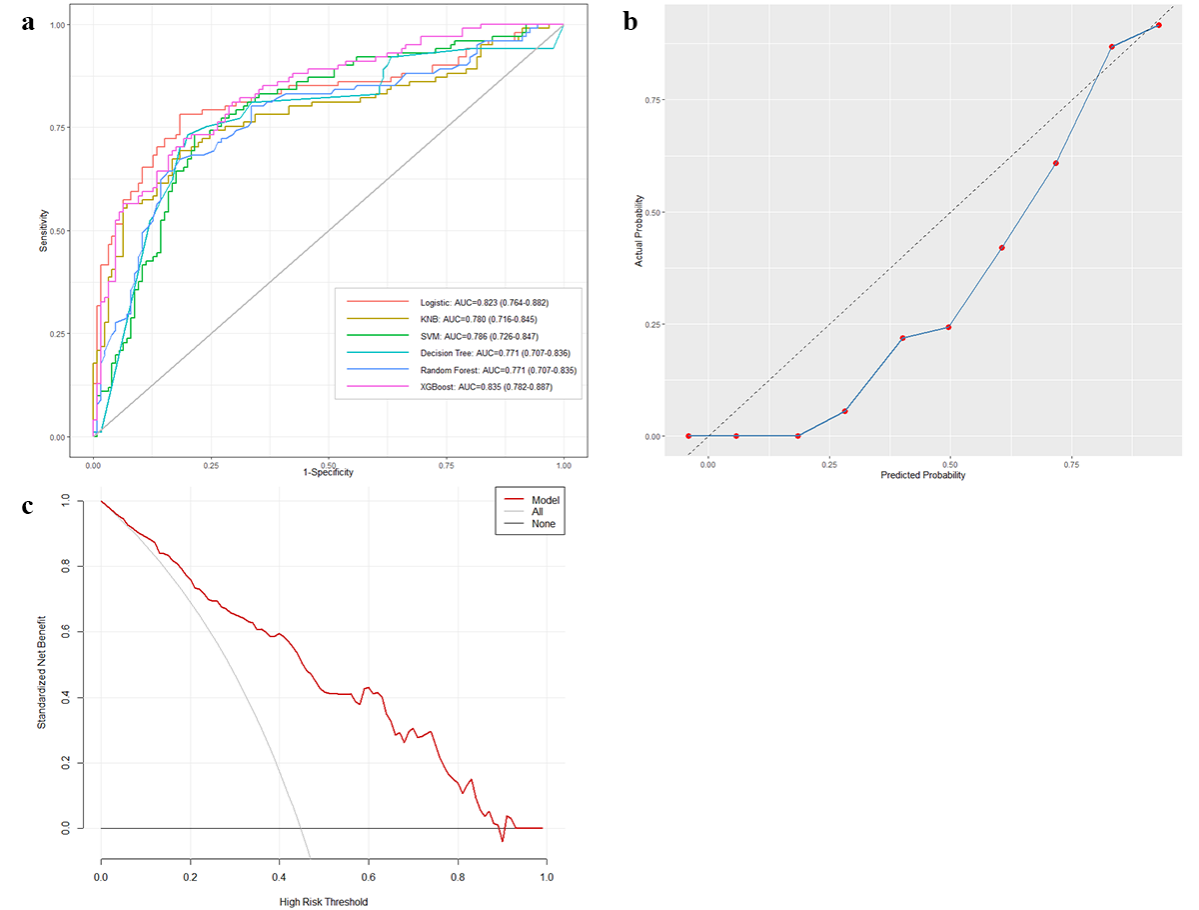
(a) Comparison of AUC values of the 6 models in the external validation set and (b) the calibration curve and (c) the DCA curve of the XGBoost model. AUC: area under the curve; DCA: decision curve analysis; KNB: kernel naive Bayes; SVM: support vector machine; XGBoost: extreme gradient boosting.

Clinical data from 226 critically ill individuals with CHD, admitted to the Second Affiliated Hospital of Zhengzhou University between January 1, 2021, and August 1, 2024, were collected and the patients included in the external validation group. Their baseline characteristics are provided in Table S4 in Multimedia Appendix 2. As shown in Figure 4a, the XGBoost model in the external validation set exhibited the highest AUROC of 0.835, similar to its performance in the testing cohort. Furthermore, the calibration curve demonstrated good calibration (Figure 4b), and the DCA curve indicated net benefit at threshold probabilities lower than 0.9 (Figure 4c), indicating that the XGBoost model has superior generalizability and can be reliably applied to external data.

### Model Interpretation

**Figure 5:**
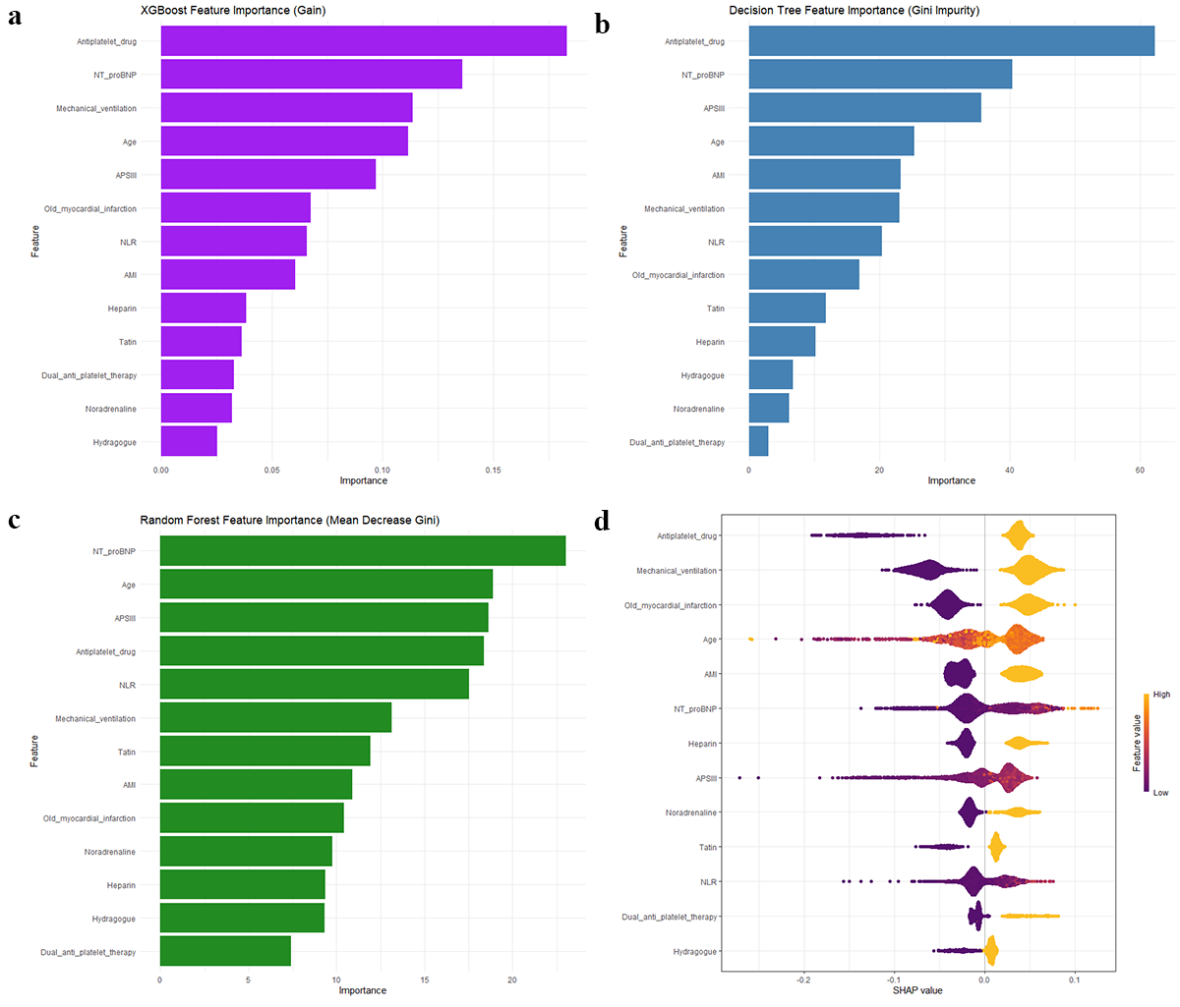
Feature importance derived from (a) XGBoost, (b) DT, and (c) RF models, along with (d) SHAP analysis summary diagram based on XGBoost. Each row represents a feature; a point represents a sample; yellow represents a high feature value; and purple represents a low feature value. A further distance from a point to the baseline SHAP value of 0 indicates a greater impact on the output. AMI: acute myocardial infarction; APSIII: acute physiology score III; NLR: neutrophil-to-lymphocyte ratio; NT-proBNP: N-terminal pro–B-type natriuretic peptide.

Our study compared the feature importance rankings of XGBoost (Figure 5a), DT (Figure 5b), and RF (Figure 5c) models, and provided global and local explanations of the predictions using SHAP values (Figure 5d), to comprehensively assess the contribution of each clinical feature to the risk of AKI. NT-proBNP ranked among the top 3 predictors in XGBoost, RF, and DT models, and its global contribution was also significant according to SHAP analysis. The SHAP dependence plot (Figure S2 in Multimedia Appendix 3) shows that as the NT-proBNP value increased, the incidence of AKI also rose, which is highly consistent with the clinical significance of NT-proBNP as a biomarker for heart failure and renal dysfunction, thereby confirming its predictive value for AKI. Age ranked high in XGBoost (fourth in gain importance) and RF models, with SHAP values indicating a positive contribution to the prediction, aligning with the clinical consensus that elderly patients are at a higher risk for AKI. Antiplatelet drugs ranked first in gain importance in the XGBoost model and in Gini impurity in the DT model; the SHAP value for high feature importance was 0.05, the SHAP value for low feature importance reached –0.2. This suggests that the use of antiplatelet drugs has a limited contribution to the promotion of AKI, while patients not using them have a lower risk of AKI. Mechanical ventilation ranked third in the XGBoost model, with its global SHAP contribution indicating a significant positive correlation with AKI risk, which supports the potential mechanisms of iatrogenic renal injury in critically ill patients. APSIII ranked third in both RF and DT models, and its SHAP dependence plot (Multimedia Appendix 3) suggests that when APSIII exceeds 40, the incidence of AKI significantly increases, reflecting the stable predictive value of composite physiological indices in AKI risk prediction.

**Figure 6:**
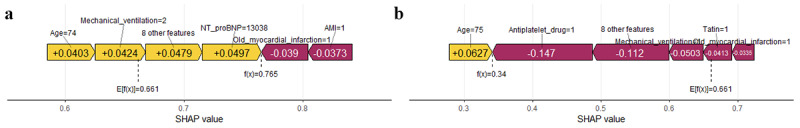
SHAP force plot of (a) a patient with AKI and (b) a patient without AKI. Features contributing to an increase in the predicted value are shown in yellow, whereas those contributing to a decrease are shown in red. The length of the arrows represents the magnitude of each feature’s influence on the model output. The scale on the x-axis indicates the extent to which each feature increases or decreases the predicted value. AKI: acute kidney injury; AMI: acute myocardial infarction; NT-proBNP: N-terminal pro–B-type natriuretic peptide; SHAP: Shapley Additive Explanation.

To intuitively demonstrate the consistency between the model’s decision logic and clinical-pathological mechanisms, this study selected 2 typical AKI risk prediction cases for SHAP force plot analysis (Figure 6). The SHAP analysis of individual samples reveals how each variable influences the model’s decision-making. As shown in Figure 6a, age, NT-proBNP, and mechanical ventilation usage contributed the highest positive predictive values, while the absence of AMI and old MI reduced the risk of AKI. In Figure 6b, the age of 75 years increased the risk of AKI, and the absence of antiplatelet drugs and mechanical ventilation usage significantly reduced the risk of AKI. These variables are all among the top 6 predictive features for AKI in the XGBoost model.

## Discussion

### Principal Findings

This study, based on data from the MIMIC-IV database and the Second Affiliated Hospital of Zhengzhou University, is the first to develop and externally validate an ML model for predicting AKI in critically ill patients with CHD. By incorporating 13 clinical variables, 6 ML algorithms were systematically compared. The results demonstrated that the XGBoost model exhibited superior predictive performance in both the internal testing set (AUROC=0.765, 95% CI 0.731-0.800) and the external validation cohort (AUROC=0.850, 95% CI 0.731-0.800). Calibration curve analysis and DCA further confirmed the model’s excellent calibration and wide range of clinically beneficial threshold probabilities (0.20-0.95). External validation also confirmed the model’s net clinical benefit across threshold probabilities below 0.9, underscoring its strong generalizability.

In model development, we used a feature selection strategy that combined LASSO regression with VIF analysis, consistent with methodologies adopted in previous studies [20]. We ultimately included13 variables and applied tree-based feature importance rankings, along with SHAP analysis, to enhance model interpretability. Our findings identified antiplatelet therapy, NT-proBNP, age, mechanical ventilation, and APSIII as the 5 most influential predictors, all of which were clinically plausible. Notably, NT-proBNP ranked among the top 3 variables in importance across the XGBoost, RF, and DT models and contributed significantly to AKI prediction, as indicated by SHAP values—an observation well aligned with its known pathophysiological role. As a sensitive biomarker of cardiorenal syndrome, NT-proBNP is cleared by glomerular filtration and can reflect early declines in the glomerular filtration rate (GFR) during the initial stages of AKI [21,22]. This finding supports the mechanistic evidence reported by Wang et al [23], who observed elevated NT-proBNP levels in patients with postoperative AKI following cardiac surgery. In addition, Liu et al [24] reported that preoperative NT-proBNP levels can more effectively predict postoperative AKI in patients undergoing noncardiac surgery, further corroborating the prognostic utility of NT-proBNP.

Given that our study cohort consisted of patients with CHD, we included relevant pharmacological agents in the predictive model. Results revealed that antiplatelet therapy ranks first in importance in both the XGBoost and DT models. SHAP analysis indicated that the risk of AKI is lowest among patients not receiving antiplatelet agents. Although monotherapy did not significantly increase AKI risk, dual-antiplatelet therapy was associated with a markedly elevated probability of AKI. Concerning how antiplatelet medications affect renal function, a Cochrane systematic review [25] showed that the risk of renal dysfunction in users of antiplatelet drugs is not high. Another systematic review indicated that in patients with chronic kidney disease (CKD), antiplatelet medication did not reduce the eGFR drop rate (−0.15 mL/1.73m²/year, 95% CI −0.89 to 1.20, I²=40.8%) and affected renal failure events (odds ratio [OR] 0.87, 95% CI 0.32-1.55) [26]. However, retrospective case-control research by Fored et al [27] on the correlation of analgesic use with renal function revealed that in subjects who rarely used acetaminophen, frequent aspirin use was linked to a 2.5-fold increase in the likelihood of chronic renal failure compared to nonusers, with the relative risk increasing as the cumulative lifetime dose increased. Similarly, Bonet-Monné et al [28] noted a decrease in renal function associated with the use of nonsteroidal anti-inflammatory drugs (NSAIDs), analgesics, and antiplatelet drugs, which aligns with our findings.

As a core indicator for assessing disease severity in critically ill patients, APSIII systematically quantifies the extent of systemic pathophysiological derangements by integrating 12 acute physiological parameters, thereby revealing its close association with the risk of AKI. In this study, the SHAP dependence plot demonstrated a marked increase in AKI incidence when the APSIII exceeded 40. This threshold effect may be attributable to the severe hemodynamic instability reflected by high APSIII values [29]. In addition, the SOFA score, a commonly used tool in ICUs, has also been widely applied for predicting AKI in critically ill patients [30]. Although originally developed for sepsis, the SOFA score has been adopted by numerous cardiovascular studies as a quantitative measure of organ dysfunction [31-33]. In our cohort, the median SOFA score was 4.00 (IQR 2.00-6.00), suggesting that a considerable proportion of patients may have been at risk for infection. Notably, although the SOFA score was not selected as a feature variable in the modeling process, NLR, an established inflammatory marker, was selected by LASSO regression and incorporated into the model. This highlights the potential role of infection in the development of AKI among critically ill patients. For example, systemic inflammatory responses may lead to tubular epithelial injury and hemodynamic disturbances that impair renal perfusion, thereby triggering the onset of AKI.

Furthermore, our study revealed that advancing age and the use of mechanical ventilation also contribute to a higher incidence of AKI. Elderly adults represent a particularly vulnerable population for AKI, as the GFR naturally declines with age and the renal functional reserve diminishes, rendering them more susceptible to AKI under stress conditions. According to the SHAP dependence plot, patients between the ages of 70 and 80 years are at the highest risk for developing AKI. Retrospective studies have shown that younger females are less likely to develop postoperative AKI; however, the incidence increases progressively with age [34]. An ML model designed for predicting postoperative AKI similarly identified age as a critical predictor [35]. The use of mechanical ventilation has likewise been associated with an elevated risk of AKI, a finding consistently supported by many studies. Patients who need invasive mechanical ventilation have a threefold higher risk of AKI, according to a high-quality systematic review and meta-analysis, with no decrease in risk despite adjustments in the tidal volume or positive end-expiratory pressure (PEEP) settings [36].

### Comparison With Previous Work

Forecasting AKI in individuals in the ICU has long been a focus in intensive care medicine. In recent years, multiple studies have developed ML models for AKI prediction. For example, Yue et al [37] developed a predictive model for AKI in patients with sepsis, Cho et al [38] established an AKI prediction model applicable to patients in general wards, and Zhou et al [39] established a forecasting model for AKI in the cohort with acute respiratory distress syndrome (ARDS) [39], incorporating 12 predictive variables, with XGBoost emerging as the ideal model, which aligns with our findings. Tseng et al [40] used 94 preoperative and intraoperative features to create a prediction model for cardiac surgery–associated AKI (CSA-AKI), where the RF model realized the highest AUROC (0.839, 95% CI 0.772-0.898) and was identified as the best model. They also used SHAP summary and dependence plots to explain the model, a similar approach to ours. Some AKI-forecasting models have been constructed for cardiovascular disease, but these primarily focus on predicting AKI following cardiac surgery. For instance, Kuno et al [41] built one for AKI after PCI [41], and Sun et al [42] predicted contrast-induced kidney injury in patients with AMI. However, patients with severe CHD in the ICU typically receive conservative treatment rather than surgical intervention. Thus, existing predictive models do not apply to these patients. At present, no forecasting models are designed for AKI in the patients with CHD in the ICU. Accordingly, we developed a dedicated predictive model for AKI among patients with CHD in the ICU, using the MIMIC-IV database. To the best of our knowledge, this is the first model specifically designed for the entire population of critically ill patients with CHD. Compared with previously established models limited to single clinical scenarios, our tool offers improved support for clinicians in identifying high-risk patients within complex clinical settings.

### Strengths

The strength of ML is in integrating diverse data types and providing personalized treatment recommendations for patients. This study included data from 2 centers: the BIDMC and the Second Affiliated Hospital of Zhengzhou University. The data were rich and multidimensional, providing a meaningful representation of real-world clinical practice. Additionally, new variables not included in previous models, such as NT-proBNP and NLR, along with additional categorical variables, were incorporated with the aim of improving the model’s performance. Moreover, the feature variables in our study differ from those in previous research. We used 13 feature variables to construct the model, including 4 continuous variables and 9 categorical variables. Categorical variables predominated in our model; however, in existing AKI prediction models, most selected variables are continuous. This discrepancy may be attributed to the fact that in addition to laboratory indicators, 25 binary categorical variables were incorporated, whereas previous studies have predominantly focused on laboratory indicators and other continuous variables, often lacking detailed data on comorbidities and medication use. Our results suggest that these categorical variables may have higher predictive value, indicating that future predictive models incorporating more categorical variables could potentially enhance predictive performance.

### Limitations

First, as a retrospective study based on the MIMIC-IV database, the substantial missing data for several key variables, such as contrast agent dosage, infection markers (eg, procalcitonin and C-reactive protein), and cardiac biomarkers, may have led to the loss of potentially valuable predictive information related to contrast-induced nephropathy, AMI, and sepsis-associated AKI. This feature selection bias could have adversely impacted the model’s ability to accurately identify AKI. Second, although hyperparameters were optimized using a grid search approach, the inclusion of 13 feature variables may have contributed to overfitting in certain models, such as the SVM and DT classifiers. Third, the external validation dataset was small, which may impact the external validity of the model. Therefore, further research involving larger sample sizes and multiple centers is necessary. Fourth, multiple imputation was used to estimate variables with less than 20% missing data, which may have introduced deviations from the true values. Additionally, this study aimed to provide a broadly applicable AKI risk assessment model for all critically ill patients with CHD—a clinical context in which the etiology of AKI is often initially unclear. However, due to the limited size of the external validation cohort, we did not perform more refined subgroup analyses to develop prediction models tailored to patients with specific comorbidities, which may have obscured potential heterogeneity across subpopulations with CHD.

### Future Work

This study established the first predictive model for AKI encompassing the entire population of critically ill patients with CHD. However, its generalizability requires further confirmation through external validation in multicenter settings. We plan to integrate multicenter data from both CCUs and general ICUs to expand the sample size of the external validation cohort, thereby providing adequate statistical power for subsequent subgroup analyses. In the future, we intend to conduct collaborative multicenter studies and perform more granular stratification of the target population, for instance, critically ill patients with CHD complicated by MI and those undergoing PCI. These efforts aim to develop tailored AKI prediction models specific to different comorbid conditions, further addressing current gaps in our research. Moreover, we will develop an interactive explanatory interface to enable a closed-loop decision support system—from risk alerting to intervention recommendations. In parallel, we will design a user-friendly interface, formulate detailed implementation guidelines, and initiate pilot projects to evaluate the model’s practical performance and user experience.

### Conclusion

In conclusion, ML models can be a trustworthy tool for forecasting AKI in individuals with severe CHD. Across tested models, the XGBoost model demonstrated the best performance. It can help physicians identify patients with CHD who are at risk of AKI early, allowing for prompt therapies to lower mortality and enhance prognosis.

## Appendix

Multimedia Appendix 1 Variance inflation factors (VIFs) of variables and model parameters.

Multimedia Appendix 2 Baseline characteristics of the test set and the external validation set.

Multimedia Appendix 3 Variable selection based on least absolute shrinkage and selection operator (LASSO) regression and Shapley Additive Explanation (SHAP) dependence plot of the extreme gradient boosting (XGBoost) model.

## Abbreviations

ACS: acute coronary syndrome
AI: artificial intelligence
AKI: acute kidney injury
ALT: alanine aminotransferase
AMI: acute myocardial infarction
APSIII: acute physiology score III
AST: aspartate aminotransferase
AUROC: area under the receiver operating characteristic curve
BIDMC: Beth Israel Deaconess Medical Center
BUN: blood urea nitrogen
CAG: coronary angiography
CCU: coronary care unit
CHD: coronary heart disease
CKMB: creatine kinase isoenzymes
DCA: decision curve analysis
DT: decision tree
EHR: electronic health record
GFR: glomerular filtration rate
ICD-10: International Classification of Diseases, 10th Revision
ICD-9: International Classification of Diseases, 9th Revision
ICU: intensive care unit
INR: international normalized ratio
IRB: Institutional Review Board
LASSO: least absolute shrinkage and selection operator
LR: logistic regression
MI: myocardial infarction
MIMIC-IV: Medical Information Mart for Intensive Care IV
MIT: Massachusetts Institute of Technology
ML: machine learning
NB: naive Bayes
NLR: neutrophil-to-lymphocyte ratio
NT-proBNP: N-terminal pro–B-type natriuretic peptide
PCI: percutaneous coronary intervention
PT: prothrombin time
PTT: partial thromboplastin time
RBC: red blood cell
RDW: red blood cell distribution width
RF: random forest
ROC: receiver operating characteristic
SCr: serum creatini
SHAP: Shapley Additive Explanation
SOFA: sequential organ failure assessment
SVM: support vector machine
VIF: variance inflation factor
WBC: white blood cell
XGBoost: extreme gradient boosting
