# Supplementary material for: Machine Learning for the Prediction of Acute Kidney Injury in Critically Ill Patients With Coronary Heart Disease: Algorithm Development and Validation
Source: JMIR Med Inform. 2025 May 28;13:e72349. doi: 10.2196/72349 (PMC12159552; doi:10.2196/72349)
Supplement: Multimedia Appendix 1 [file medinform_v13i1e72349_app1.docx]

**Multimedia Appendix 1** Variance Inflation Factors of Variables and Model Parameters

**Table S1** Variance inflation factor of feature variables

| **Variables** | VIF |
| --- | --- |
| Age | 1.051517 |
| Mechanical_ventilation | 1.230826 |
| NT_proBNP | 1.085124 |
| NLR | 1.038644 |
| AMI | 1.144294 |
| Old_myocardial_infarction | 1.147919 |
| Antiplatelet_drug | 1.309595 |
| Dual_anti_platelet_therapy | 1.104072 |
| Heparin | 1.057010 |
| Hydragogue | 1.146318 |
| Noradrenaline | 1.154277 |
| Tatin | 1.245497 |
| APSIII | 1.127907 |

**Table S2** Optimal hyperparameters for each model configuratione

| Model | Hyperparameters |
| --- | --- |
| Logistic Regression | family = binomial, method = "glm.fit" epsilon = 1e-8, maxit = 25, trace = FALSE |
| Naive Bayes | laplace = 3, threshold = 0.001, eps = 0 |
| Support Vector Machine | kernel = "radial", cost = 1, gamma = 0.077, degree = 3, coef0 = 0 |
| Decision Trees | minsplit =20, maxdepth =7, xval = 10, cp = 0.001 |
| Random Forest | ntree = 300, mtry=1, importance=TRUE, proximity=TRUE |
| Extreme Gradient Boosting | max_depth=3,eta=0.1, nround=100, gamma=0.6, colsample_bytree=0.6 ,min_chile_weight=1, sbsample=1 |
