# Supplementary material for: Machine Learning for the Prediction of Acute Kidney Injury in Critically Ill Patients With Coronary Heart Disease: Algorithm Development and Validation
Source: JMIR Med Inform. 2025 May 28;13:e72349. doi: 10.2196/72349 (PMC12159552; doi:10.2196/72349)
Supplement: Multimedia Appendix 2 [file medinform_v13i1e72349_app2.docx]

**Multimedia Appendix 2** Baseline Characteristics of the Test Set and External Validation Set

**Table S3.** Baseline characteristics of test cohort

| **Variables** | | **Total (n=2711)** | **Test(n=814)** | | |
| --- | --- | --- | --- | --- | --- |
|  |  |  | **Non-AKI (n=259)** | **AKI^a^ (n=555)** | **P-value** |
| Age (year), median (IQR) | | 71.0 [62.0;79.0] | 70.0 [59.0;79.0] | 72.0 [63.0;80.0] | .07 |
| BMI(kg/m2), median (IQR) | | 29.1 [25.5;34.0] | 29.8 [26.0;34.0] | 29.0 [25.5;33.4] | .26 |
| **Gender, n(%)** | |  |  |  | .002 |
|  | Male | 144 (55.6) | 144 (55.6) | 346 (62.3) |  |
|  | Female | 115 (44.4) | 115 (44.4) | 209 (37.7) |  |
| **Marital Status, n(%)** | |  |  |  | .22 |
|  | Single | 60 (23.2) | 60 (23.2) | 124 (22.3) |  |
|  | Divorced/Widowed | 67 (25.9) | 67 (25.9) | 116 (20.9) |  |
|  | Married | 1435 (52.9) | 132 (51) | 315 (56.8) |  |
| **Race, n(%)** | |  |  |  | .06 |
|  | White | 179 (69.1) | 179 (69.1) | 420 (75.7) |  |
|  | No White | 80 (30.9) | 80 (30.9) | 135 (24.3) |  |
| HeartRate(beats/minute), median (IQR) | | 83.0 [73.0;96.0] | 85.0 [74.0;102] | 84.0 [73.0;96.0] | .14 |
| Mechanical ventilation, n(%) | | 1517 (55.9) | 89 (34.4) | 356 (64.1) | <.001 |
| RespiratoryRate(beats/minute), median (IQR) | | 18.0 [15.0;22.0] | 18.0 [15.0;23.0] | 18.0 [14.0;22.0] | .57 |
| **Laboratory values,** **median (IQR)** | |  |  |  |  |
|  | Albumin(g/dL) | 3.60 [3.10;4.00] | 3.60 [3.10;4.00] | 3.50 [3.00;3.90] | .004 |
|  | ALT^b^(IU/L) | 20.0 [14.0;37.0] | 20.0 [14.0;37.0] | 22.0 [14.0;39.5] | .41 |
|  | Anion gap(mmol/L) | 14.0 [12.0;17.0] | 14.0 [12.0;17.0] | 14.0 [12.0;17.0] | .95 |
|  | AST^c^(IU/L) | 28.0 [19.0;41.0] | 28.0 [19.0;41.0] | 29.0 [20.0;50.0] | .02 |
|  | Bicarbonate(mmol/L) | 24.0 [21.0;26.0] | 24.0 [21.0;26.0] | 23.0 [21.0;26.0] | .25 |
|  | Bilirubin Total(mg/dL) | 0.50 [0.30;0.80] | 0.50 [0.30;0.80] | 0.60 [0.35;0.90] | .01 |
|  | Potassium(mEq/L) | 4.20 [3.80;4.60] | 4.20 [3.80;4.60] | 4.20 [3.80;4.60] | .49 |
|  | Sodium(mEq/L) | 139 [136;141] | 139 [136;141] | 138 [136;141] | .52 |
|  | NT-proBNP(pg/mL) | 1425 [414;4064] | 1425 [414;4064] | 2718 [826;8846] | <.001 |
|  | BUN^d^(mg/dL) | 20.0 [14.0;34.0] | 20.0 [14.0;34.0] | 24.0 [16.0;35.0] | .01 |
|  | Chloride（mEq/L) | 103 [100;106] | 103 [100;106] | 104 [100;108] | .47 |
|  | CKMB^e^(ng/mL) | 3.00 [2.00;7.00] | 3.00 [2.00;7.00] | 4.00 [2.00;7.00] | .32 |
|  | Hemoglobin(g/dL) | 10.6 [8.60;12.0] | 10.6 [8.60;12.0] | 10.3 [8.70;12.0] | .44 |
|  | INR^f^ | 1.20 [1.10;1.50] | 1.20 [1.10;1.50] | 1.30 [1.10;1.50] | .08 |
|  | Lactate（mmol/L) | 1.60 [1.20;2.45] | 1.60 [1.20;2.45] | 1.70 [1.20;2.40] | .62 |
|  | Lymphocyte count(10^9/L) | 1.28 [0.84;1.88] | 1.28 [0.84;1.88] | 1.22 [0.81;1.79] | .20 |
|  | Lymphocytes(%) | 15.0 [9.30;23.0] | 15.0 [9.30;23.0] | 13.4 [8.00;20.7] | .03 |
|  | Neutrophil count(10^9/L) | 5.92 [3.95;9.08] | 5.92 [3.95;9.08] | 6.76 [4.44;9.96] | .03 |
|  | Neutrophils(%) | 74.0 [65.3;82.0] | 74.0 [65.3;82.0] | 77.1 [68.0;84.0] | .005 |
|  | Plt^g^(K/uL) | 197 [150;246] | 197 [150;246] | 174 [136;232] | .007 |
|  | NLR^h^ | 4.70 [2.70;8.10] | 4.70 [2.70;8.10] | 5.30 [3.10;9.80] | .01 |
|  | PT^i^(seconds) | 13.7 [12.2;16.2] | 13.7 [12.2;16.2] | 14.1 [12.4;17.0] | .14 |
|  | PTT^j^(seconds) | 30.5 [27.1;35.8] | 30.5 [27.1;35.8] | 31.9 [27.8;38.5] | .03 |
|  | RBC^k^(m/uL) | 3.58 [2.98;4.10] | 3.58 [2.98;4.10] | 3.47 [2.93;3.96] | .14 |
|  | RDW^l^(%) | 14.6 [13.5;15.8] | 14.6 [13.5;15.8] | 14.6 [13.7;15.9] | .83 |
|  | serum creatinine (mg/dL) | 1.00 [0.70;1.50] | 1.00 [0.70;1.50] | 1.10 [0.80;1.60] | .006 |
|  | WBC^m^(K/uL) | 10.3 [7.25;13.4] | 10.3 [7.25;13.4] | 10.4 [7.50;14.5] | .25 |
| **Comorbidity, n（%）** | |  |  |  |  |
|  | Acute myocardial infarction | 91 (35.1) | 91 (35.1) | 255 (45.9) | .005 |
|  | Atrial fibrillationp | 138 (53.3) | 138 (53.3) | 338 (60.9) | .04 |
|  | Diabetes | 127 (49) | 127 (49) | 329 (59.3) | .008 |
|  | Heatr failure | 199 (76.8) | 199 (76.8) | 478 (86.1) | .001 |
|  | Hypertension | 232 (89.6) | 232 (89.6) | 532 (95.9) | .001 |
|  | Old myocardial infarction | 98 (37.8) | 98 (37.8) | 287 (51.7) | <.001 |
| **Drugs, n（%）** | |  |  |  |  |
|  | ACEI_ARB | 113 (40.6) | 113 (40.6) | 281 (49.6) | .06 |
|  | Antibiotic | 163 (62.9) | 163 (62.9) | 442 (79.6) | <.001 |
|  | Antiplatelet drug | 166 (64.1) | 166 (64.1) | 488 (87.9) | <.001 |
|  | Aspirin | 129 (49.8) | 129 (49.8) | 405 (73) | <.001 |
|  | Clopidogrel | 30 (11.6) | 30 (11.6) | 144 (25.9) | <.001 |
|  | Dual_anti_platelet_therapy | 22 (8.5) | 22 (8.5) | 125 (22.5) | <.001 |
|  | Dobutamine | 7 (2.7) | 7 (2.7) | 23 (4.1) | .41 |
|  | Dopamine | 10 (3.9) | 10 (3.9) | 26 (4.7) | .72 |
|  | Epinephrine | 19 (7.3) | 19 (7.3) | 80 (14.4) | .006 |
|  | Heparin | 57 (22) | 57 (22) | 222 (40) | <.001 |
|  | Hydragogue | 177 (68.3) | 177 (68.3) | 479 (86.3) | <.001 |
|  | Noradrenaline | 52 (20.1) | 52 (20.1) | 205 (36.9) | <.001 |
|  | Two vasoactive drugs | 15 (5.8) | 15 (5.8) | 71 (12.8) | .004 |
|  | Three vasoactive drugs | 2 (0.8) | 2 (0.8) | 12 (2.2) | .25 |
|  | Tatin | 172 (66.4) | 172 (66.4) | 473 (85.2) | <.001 |
| CAG^n^, n（%） | | 152 (5.6) | 8 (3.1) | 36 (6.5) | .07 |
| PCI^o^, n（%） | | 76 (2.8) | 4 (1.5) | 17 (3.1) | .30 |
| APSIII^p^, mean (SD) | | 43.5 (17.0) | 40.1 (16.2) | 45.2 (16.7) | <.001 |
| SOFA^q^, median (IQR) | | 4.00 [2.00;6.00] | 3.00 [2.00;5.00] | 5.00 [3.00;7.00] | <.001 |

^a^AKI: acute kidney injury; ^b^ALT: alanine aminotransferase; ^c^AST: aspartate aminotransferase; ^d^BUN: blood urea nitrogen; ^e^CKMB: creatine kinase isoenzymes; ^f^INR: international normalized ratio; ^g^PLT: blood platelet; ^h^NLR: neutrophil/lymphocyte ratio; ^i^PT: prothrombin time; ^j^PTT: partial thromboplastin time; ^k^RBC: red blood cells; ^l^RDW: Red blood cell distribution width; ^m^WBC: white blood cells; ^n^CAG:coronary angiography; ^o^PCI:percutaneous coronary intervention; ^p^APSIII: acute physiology score III; ^q^SOFA: sequential organ failure assessment.

**Table S4.** Baseline characteristics of the external validation cohort

| **Variables** | | **Total (n=226)** | **Non-AKI(n=125)** | **AKI^a^(n=101)** | **P-value** |
| --- | --- | --- | --- | --- | --- |
| Age (year),mean (SD) | | 66.2 (12.2) | 65.4 (12.2) | 67.3 (12.2) | .27 |
| Male，n（%） | | 148 (65.5) | 87 (69.6) | 61 (60.4) | .19 |
| HeartRate(beats/minute)，mean (SD) | | 92.66(27.82) | 86.45(25.82) | 98.19(25.78) | <.001 |
| Mechanical ventilation, n（%） | | 99 (43.8) | 28 (22.4) | 71 (70.3) | <.001 |
| **Laboratory values, median (IQR)** | |  |  |  |  |
|  | NT-proBNP(pg/mL) | 3949 [1230;8478] | 1557 [741;5010] | 6950 [3882;13015] | <.001 |
|  | serum creatinine (mg/dL) | 1.00 [0.70;1.30] | 0.80 [0.60;0.90] | 1.30 [1.00;1.90] | <.001 |
|  | WBC(K/uL) | 12.0 [8.98;15.4] | 11.2 [8.41;14.2] | 12.9 [10.5;18.5] | .001 |
|  | Neutrophil count(10^9/L) | 9.97 [7.44;13.9] | 9.23 [6.38;12.2] | 11.1 [8.69;16.1] | <.001 |
|  | Lymphocyte count(10^9/L) | 1.09 [0.69;1.53] | 1.15 [0.77;1.54] | 1.08 [0.64;1.43] | .12 |
|  | NLR^b^ | 9.40 [5.43;17.2] | 8.40 [4.40;15.0] | 10.9 [6.50;19.3] | .002 |
| Bilirubin Total(mg/dL), mean (SD) | | 1.12(0.66) | 1.13(0.66) | 1.21(0.88) | .87 |
| **Comorbidity, n（%）** | |  |  |  |  |
|  | AMI^c^ | 165 (73.0) | 89 (71.2) | 76 (75.2) | .59 |
|  | Atrial fibrillationp | 31 (13.7) | 11 (8.80) | 20 (19.8) | .03 |
|  | Diabetes | 71 (31.4) | 35 (28.0) | 36 (35.6) | .28 |
|  | Heatr failure | 109 (48.2) | 43 (34.4) | 66 (65.3) | <.001 |
|  | Hypertension | 113 (50.0) | 62 (49.6) | 51 (50.5) | .99 |
|  | Old myocardial infarction | 30 (13.3) | 6 (4.80) | 24 (23.8) | <.001 |
| **Drugs, n（%）** | |  |  |  |  |
|  | Antiplatelet drug | 196 (86.7) | 109 (87.2) | 87 (86.1) | .97 |
|  | Aspirin | 183 (81.0) | 102 (81.6) | 81 (80.2) | .92 |
|  | Clopidogrel | 189 (83.6) | 106 (84.8) | 83 (82.2) | .73 |
|  | Dual_anti_platelet_therapy | 175 (77.4) | 98 (78.4) | 77 (76.2) | .82 |
|  | ACEI_ARB | 37 (16.4) | 23 (18.4) | 14 (13.9) | .46 |
|  | Tatin | 164 (72.6) | 96 (76.8) | 68 (67.3) | .15 |
|  | Antibiotic | 115 (50.9) | 56 (44.8) | 59 (58.4) | .06 |
|  | Noradrenaline | 78 (34.5) | 18 (14.4) | 60 (59.4) | <.001 |
|  | Epinephrine | 22 (9.73) | 4 (3.20) | 18 (17.8) | .001 |
|  | Heparin | 45 (19.9) | 16 (12.8) | 29 (28.7) | .005 |
|  | Hydragogue | 89 (39.4) | 36 (28.8) | 53 (52.5) | <.001 |
| APSIII^d^，mean (SD) | | 26.20(20.62) | 16.04(16.72) | 39.19(17.69) | <.001 |
| PCI^e^, n（%） | | 129 (57.1) | 83 (66.4) | 46 (45.5) | .003 |

^a^AKI: acute kidney injury; ^b^NLR: neutrophil/lymphocyte ratio; ^c^acute myocardial infarct; ^d^APSIII: acute physiology score III; ^e^PCI:percutaneous coronary intervention
