# Supplementary material for: Machine Learning for the Prediction of Acute Kidney Injury in Critically Ill Patients With Coronary Heart Disease: Algorithm Development and Validation
Source: JMIR Med Inform. 2025 May 28;13:e72349. doi: 10.2196/72349 (PMC12159552; doi:10.2196/72349)
Supplement: Multimedia Appendix 3 [file medinform_v13i1e72349_app3.docx]

**Multimedia Appendix 3** Variable Selection Based on LASSO Regression and SHAP Dependence Plot of the XGBoost Model

**
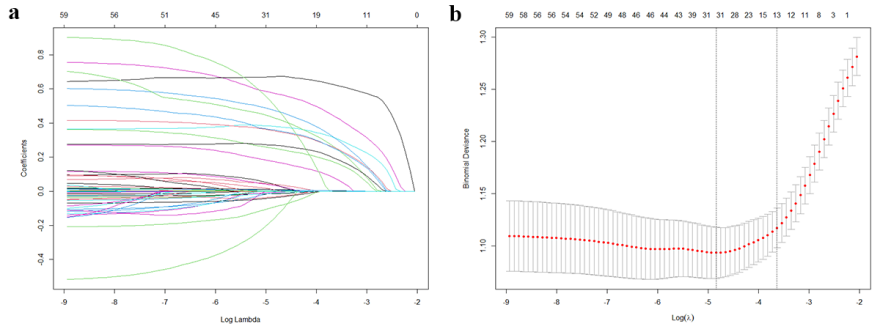
**

**Figure S1** Variable screening based on Lasso regression. (a) The changing characteristics of variable coefficients; (b) The process of selecting the optimal value of parameter λ in the Lasso regression model through cross-validation method.


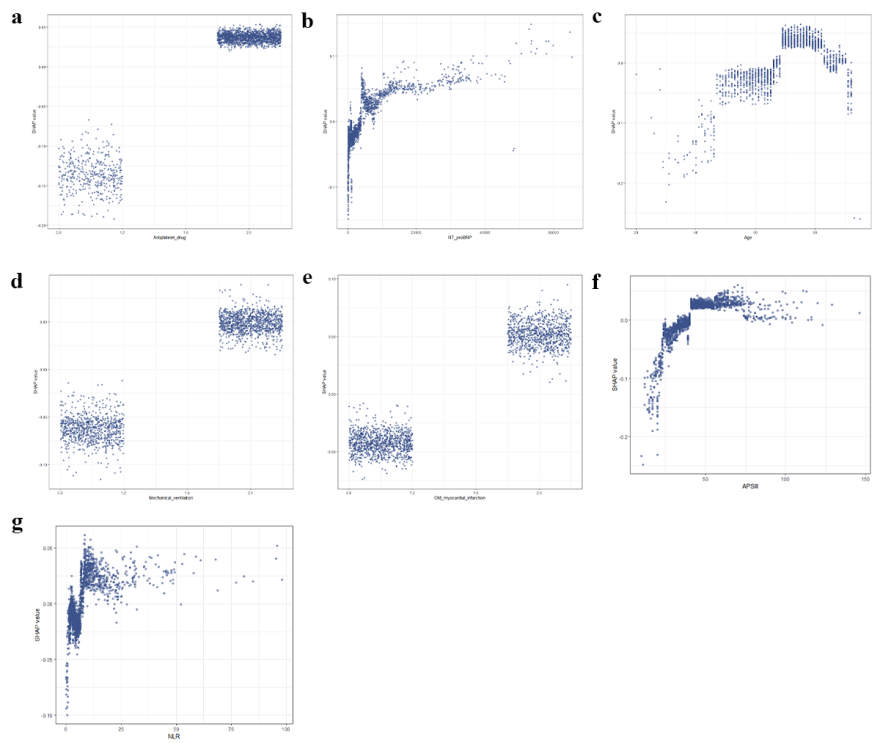


**Figure S2** SHAP dependence plot of the XGBoost model. The SHAP dependence plot shows how a single feature affects the output of the XGBoost prediction model. SHAP values for specific features exceed zero, representing an increased risk of acute kidney injury development.
